# UKSSL: Underlying Knowledge Based Semi-Supervised Learning for Medical Image Classification

**DOI:** 10.1109/OJEMB.2023.3305190

**Published:** 2023-08-15

**Authors:** Zeyu Ren, Xiangyu Kong, Yudong Zhang, Shuihua Wang

**Affiliations:** University of Leicester 4488 LE1 7RH Leicester U.K.

**Keywords:** Deep learning, self-supervised learning, medical image analysis, semi-supervised learning, image classification

## Abstract

*Goal:* Deep learning techniques have made significant progress in medical image analysis. However, obtaining ground truth labels for unlabeled medical images is challenging as they often outnumber labeled images. Thus, training a high-performance model with limited labeled data has become a crucial challenge. *Methods:* This study introduces an underlying knowledge-based semi-supervised framework called UKSSL, consisting of two components: MedCLR extracts feature representations from the unlabeled dataset; UKMLP utilizes the representation and fine-tunes it with the limited labeled dataset to classify the medical images. *Results:* UKSSL evaluates on the LC25000 and BCCD datasets, using only 50% labeled data. It gets precision, recall, F1-score, and accuracy of 98.9% on LC25000 and 94.3%, 94.5%, 94.3%, and 94.1% on BCCD, respectively. These results outperform other supervised-learning methods using 100% labeled data. *Conclusions:* The UKSSL can efficiently extract underlying knowledge from the unlabeled dataset and perform better using limited labeled medical images.

## I. Introduction

Extracting underlying knowledge from limited labeled data is a challenging problem in machine learning, especially for medical image analysis, where annotation of medical images is costly and laborious. However, in medical image analysis, there are plenty of unlabeled or limited labeled images instead of all the images with ground-truth labels. Therefore, it is desirable to pay attention to the techniques with weak supervision or non-supervision, which can utilize unlabeled and limited labeled datasets in medical image analysis. In the last decade, machine learning has been developing a series of paradigms to deal with insufficient data annotation problems, such as semi-supervised learning [25], multi-instance learning [26], and self-supervised learning [27]. Within these paradigms, semi-supervised learning-based approaches are attractive because semi-supervised learning is able to achieve better performance than the self-supervised methods and it only needs a limited labeled dataset than the supervised methods.

Semi-supervised methods mainly consist of five types: generative methods [17], [18], consistency regularization methods [15], [16], graph-based methods [13], [14], pseudo-labeling methods [11], [12] and hybrid methods [9], [10]. One popular paradigm within the hybrid methods is combining self-supervised learning with supervised learning to construct a semi-supervised learning-based model. Our method is also based on this paradigm which constructs a contrastive learning-based model and then fine-tunes it with the limited labeled dataset.

Contrastive learning is one type of self-supervised learning, which aims to learn the fusion information of a dataset such that similar data instances are close and diverse data instances are far away from each other [8]. The most basic idea of contrastive learning is to learn semantic representations by constructing positive pairs (data points that should be similar) and negative pairs (data points that should be dissimilar). In recent years, there has been a surge of interest in contrastive learning. Some prior works like InstDisc [7], CPC [6], and CMC [5] attract lots of researchers to focus on this area. Later on, some significant works like SimCLR [28], MoCo [4], and SwAv [3] are proposed. These works introduce the momentum encoder and InfoNCE loss function, which accelerate the development of this area. Moreover, some works also focus on building the prediction task without negative pairs, such as BYOL [2] and SimSiam [1].

In this work, we employ a contrastive learning model to extract the feature map from unlabeled datasets. Subsequently, we construct a supervised learning model and fine-tune it using a limited labeled dataset. The proposed framework UKSSL achieves outstanding performance by using limited datasets compared with other state-of-the-art methods on the different medical image classification datasets: LC25000 and BCCD datasets. We observe that the self-supervised pre-training with an unlabeled dataset can get a meaningful underlying knowledge of the dataset, and fine-tuning this representation with a limited labeled dataset can achieve excellent performance on downstream tasks. We attribute this finding to exploring the robust contrastive model architectures and algorithms to enhance the performance of our framework.

**Figure 1.**
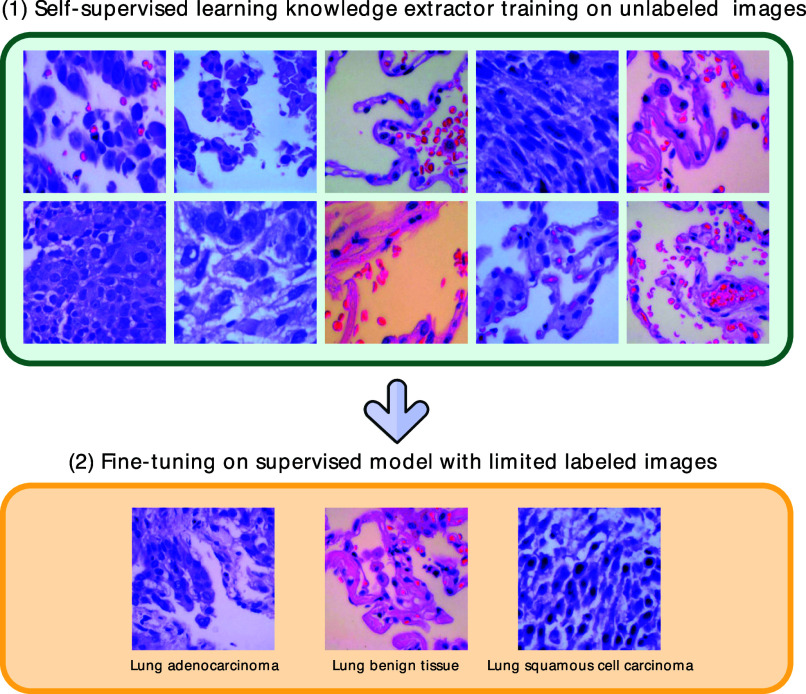
Our framework includes two components: (1) Training a knowledge extractor MedCLR on the unlabeled medical images based on the SimCLR [28]. (2) Using limited labeled medical images to fine-tune the UKMLP.

The structure of the proposed framework is presented in Fig. 1, and our key findings and contributions are as follows:

• The proposed framework UKSSL achieves excellent performance with partially labeled medical images, and it gets the best performance compared with other supervised methods.
• We propose a MedCLR, which can efficiently extract the underlying knowledge from the unlabeled dataset. These extracted representations can be used in different proxy tasks.
• We present a UKMLP, it improves the performance by using the underlying knowledge provided by the self-supervised model, which can be used with other self-supervised models.

## II. Materials and Methods

Our proposed UKSSL consists of two parts: MedCLR and UKMLP. MedCLR is inspired by SimCLR [28], which uses contrastive learning to extract underlying knowledge from unlabeled images. After pre-training the MedCLR, the second part UKMLP will utilize the underlying knowledge obtained by the MedCLR to fine-tune with limited labeled medical images. Then after finishing the entire process, the well-trained UKSSL can classify the medical images. In this section, we will introduce our MedCLR in Section II-A, then we will illustrate our UKMLP in Section II-B. Finally, the materials and experimental settings are shown in Section II-C.

### A. Contrastive Learning of Medical Visual Representations (MedCLR)

**Figure 2.**
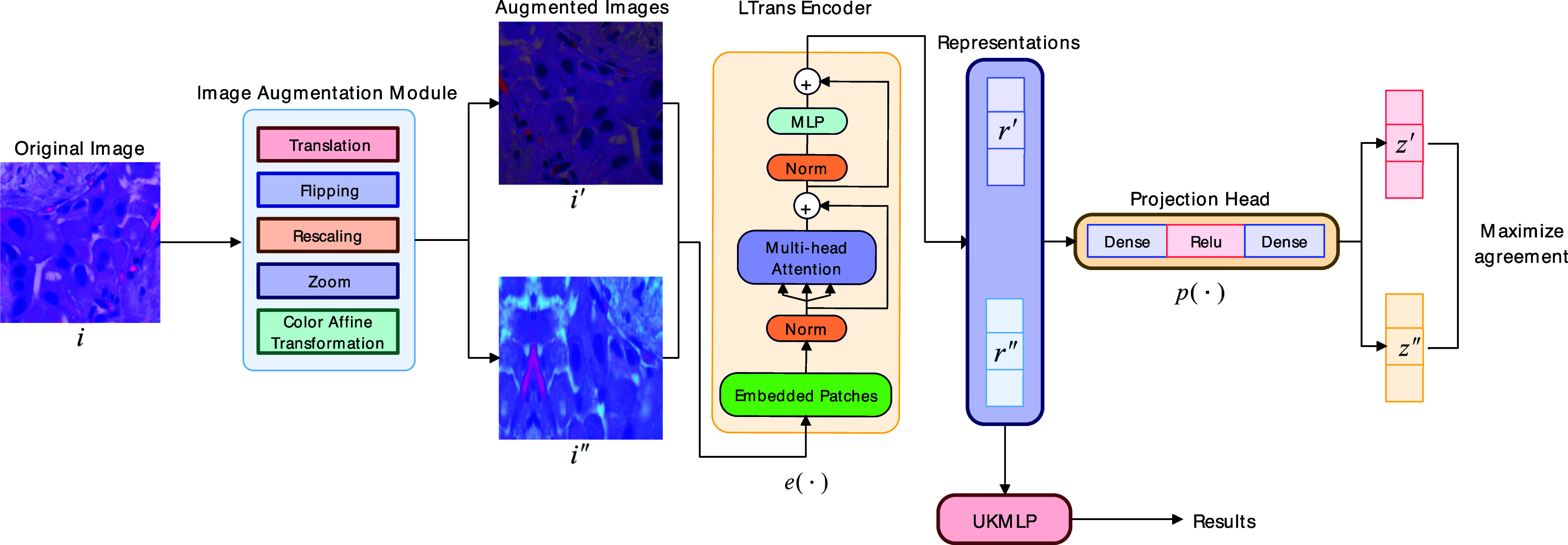
Architecture of the MedCLR. *i* denotes the original image, *$i^{\prime }$* and *$i^{\prime \prime }$* denote two different augmented images from the original image *i*, *$e(\cdot)$* denotes the encoder, and there are two representations generated by the encoder called *$i^{\prime }$* and *$i^{\prime \prime }$*, *$p(\cdot)$* denotes for the projection head and *$z^{\prime }$*, *$z^{\prime \prime }$* represents for the feature vectors from the projection head.

To learn the medical images' visual representations, we design our MedCLR, which inspires by a contrastive learning method SimCLR [28]. MedCLR extracts the semantic information by maximizing agreement [29] between the different augmented images from the same image with contrastive loss function in the underlying semantic knowledge distributions. As Fig. 2 illustrates, there are four components of MedCLR: image augmentation module, deep learning-based encoder, projection head, and a contrastive loss function NT-Xent.

*Image augmentation module* applies multiple data augmentation techniques to transform the original image *i* to two augmented images *$i^{\prime }$* and *$i^{\prime \prime }$*, the pair of *$i^{\prime }$* and *$i^{\prime \prime }$* also called positive pair in one batch of the dataset, we denote the image augmentation module as $A$. In detail, there are five data augmentation techniques: rescaling the input data to the range of $0-255$, random flipping of the images, random translation followed by the random zoom, and random color affine transformation.

*Encoder* denotes by the *$e(\cdot)$*, which can extract the semantic knowledge from the augmented images to two representations *$r^{\prime }$* and *$r^{\prime \prime }$*. In our framework, we inspire ideas from the Vision Transformer (ViT) [32] to design our light encoder architecture LTrans. It obtains representations *$r^{\prime }$* and *$r^{\prime \prime }$* as (1) shows, where the output $r^{\prime }\in \mathbb {R}^{d}$ is generated by an average pooling layer.

\begin{equation*}
r^{\prime } = e(i^{\prime }) = Encoder(i^{\prime }) \tag{1}
\end{equation*}

**Figure 3.**
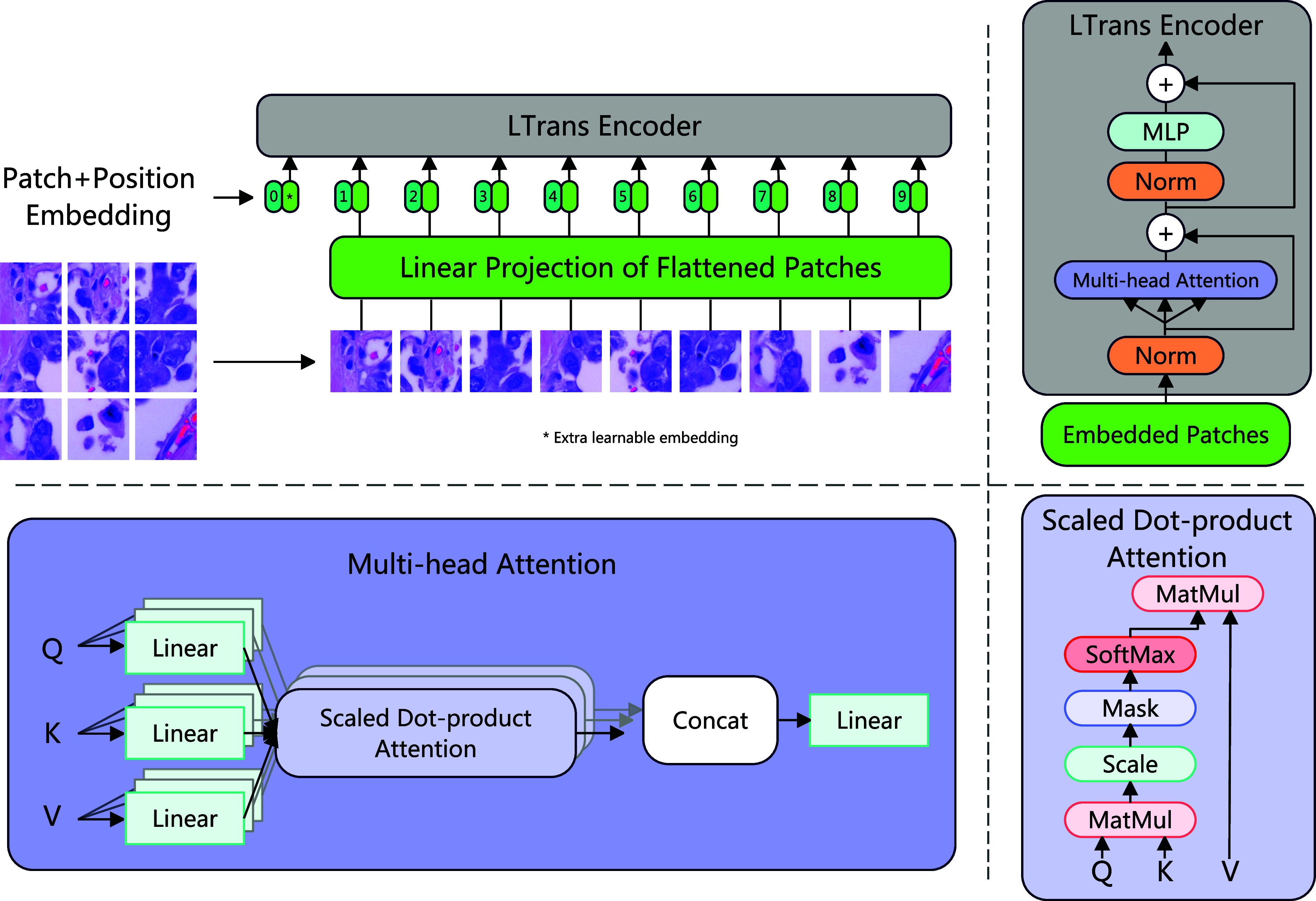
Architecture of the LTrans. Firstly, we reshape the original image into a sequence of flattened 2D patches and pass them into a linear projection with trainable parameters. Then the embedded patches pass the LTrans. The LTrans contains two normalization layers, two residual connections, a multi-head attention module, and an MLP. The details of the multi-head attention module are also provided in this figure.

The architecture of the encoder is shown in Fig. 3. Unlike the traditional Transformer, which will regard a 1D sequence of token embeddings as the input, we replace it by reshaping the image from the size $H\times W\times C$ to a sequence of flattened 2D patches with size $N\times P^{2}\cdot C$. Here, the $H$ and $W$ indicate the height and width of the original image. The $C$ represents the number of channels. The $P^{2}$ is the resolution of each image patch, and the number of patches is calculated by the (2).

\begin{equation*}
N_{patch} = (H W \mid P)^{2} \tag{2}
\end{equation*}

After reshaping from original images to patches, we map flattened patches into $D$ dimensions and pass them into a linear projection with trainable parameters. The linear projection is shown in (3), where $i_{p}$ represents the flattened 2D patches from the original image $i$. The $i_{class}$ denotes a special classification token. This is similar to the $[CLS]$ token in the BERT [33]. The output from this projection is called patch embeddings. We also use the position embeddings $e_{position}$ to keep the positional information. In detail, we use standard learnable 1D position embeddings, and after generating the position embeddings, we add the position embeddings with patch embeddings together to produce the final embedded patches $E_{0}$. Then passing embedded patches into the LTrans.

\begin{equation*}
\begin{aligned} E_{0} &= e_{position}+\left[i_{class}; i_{p}^{1} e; i_{p}^{2} e; \cdots; i_{p}^{N} e\right], \\
e &\in \mathbb {R}^{\left(P^{2} \cdot C\right) \times D}, e_{position} \in \mathbb {R}^{(N+1) \times D} \end{aligned} \tag{3}
\end{equation*}

LTrans consists of multi-head self-attention (MSA) [32] and MLP blocks [34], and we add a normalization layer before each component, also a residual connection [35] after each component. Multi-head attention [31] attracts lots of researchers to use in their models. In detail, assuming we have an input sequence $x \in \mathbb {R} ^{N \times D}$, we calculate a weighted sum over each value $V$ in the input sequence $x$, as (4) shows. The weights of attention $Attention_{mn}$ are determined by comparing the pairwise similarity of two elements within the sequence, along with their corresponding representations of query $Q^{m}$ and key $K^{n}$, as (5) shows. Finally, the self-attention $Sa$ is calculated by the (6).

\begin{align*}
[Q, K, V]&=x U_{Q K V}, \quad U_{Q K V} \in \mathbb {R}^{D \times 3 D_{h}} \tag{4}\\
{\rm Attention} &=\operatorname{softmax} \frac{Q K^{T}}{\sqrt{D_{h}}}, \quad Attention \in \mathbb {R}^{N \times N} \tag{5}\\
Sa(x) &= AttentionV \tag{6}
\end{align*}

Multihead self-attention (MSA) runs $k$ self-attention operations and projects their concatenated outputs, which is shown in (7).

\begin{align*}
\operatorname{MSA}(x)=&\left[\text{Sa}_{1}(x); \text{Sa}_{2}(x); \cdots; \text{Sa}_{k}(x)\right] U_{QKV}, \quad U_{QKV} \\
\in &\mathbb {R}^{k \cdot D_{h} \times D} \tag{7}
\end{align*}

The MLP blocks in the LTrans have two fully connected layers with GELU non-linearity. The entire process of LTrans can be described in (8) and (9).

\begin{align*}
x_{\ell }^{\prime } &=\operatorname{MSA}\left(\text{Norm}\left(x_{\ell -1}\right)\right)+x_{\ell -1}, \quad \ell =1 \ldots L \tag{8}
\\
x_{\ell }&=\operatorname{MLP}\left(\text{Norm}\left(x_{\ell }^{\prime }\right)\right)+x_{\ell }^{\prime }, \quad \ell =1 \ldots L \tag{9}
\end{align*}

*Projection head* $p(\cdot)$ is a small non-linear MLP neural network that can project the representation $r$ to another feature space $z$. As (10) shows, the $\sigma$ is a non-linear ReLU function, the $W^{(1)}$ is the weight for the encoder $e(\cdot)$, and the $W^{(2)}$ is the weight for the projection head $p(\cdot)$.

\begin{equation*}
z = p(r) = W^{(2)}\sigma (W^{(1)}r) \tag{10}
\end{equation*}

*Contrastive loss function NT-Xent* is designed to optimize the performance of the prediction task, as (11) shows. This loss function is termed by Chen et al. [28] as NT-Xent (the normalized temperature-scaled cross-entropy loss), and it has been widely used in lots of works [6], [7], [30].

As Fig. 2 shows, given a minibatch with random $N$ samples generated by the entire dataset, each image $i$ will produce two augmented images by the image augmentation module denoted by *$i^{\prime }$* and *$i^{\prime \prime }$*. This process results in $\text{2}\;N$ data samples. Within these $\text{2}\;N$ data samples, we regard two augmented images from the original image as positive pairs, and the remaining images $2(N-1)$ are negative pairs. Afterward, the two augmented images are passed into the encoder neural network $e(\cdot)$ to produce the representations $r^{\prime }$ and $r^{\prime \prime }$. Then representations are fed into the non-linear MLP projection head $p(\cdot)$ and yield feature space *$z^{\prime }$* and *$z^{\prime \prime }$* for calculations of contrastive loss.

The loss function NT-Xent between a pair of positive examples $(i^{\prime },i^{\prime \prime })$ is defined as follows:

\begin{equation*}
\begin{aligned} \ell _{i^{\prime }, i^{\prime \prime }}^{\text{NT-Xent}}=-\log \frac{\exp \left(\operatorname{sim}\left(\boldsymbol{z}^{\prime }, \boldsymbol{z}^{\prime \prime }\right) / \tau \right)}{\sum _{k=1}^{\text{2}\;N} \mathbb {1}_{[k \ne i]} \exp \left(\operatorname{sim}\left(\boldsymbol{z}_{i}, \boldsymbol{z}_{k}\right) / \tau \right)} \end{aligned} \tag{11}
\end{equation*}

where $\mathbb {1}_{[k \ne i]}$ represents an indicator function, and iff $k\ne i$, the value of the function is equal to 1. The $\tau$ is called the temperature coefficient, which can control the strength of penalties on the hard negative samples. Finally, the loss is computed across all the positive pairs.

Combining the four components mentioned above, we build the completed MedCLR, and we provide the pseudo-code of MedCLR in Algorithm 1. In this algorithm, we have inputs such as a batch of data with size $N$, constant $\tau$, encoder $e$, projection head $p$, and data augmentation module $A$. Initially, we sample a mini-batch and do the two augmentations separately. After the data is augmented, we pass the augmented images into the encoder $e(\cdot)$ and projection head $p(\cdot)$. After that, we do the pairwise similarity and calculate the loss function by updating the parameters of encoder $e(\cdot)$ and projection head $p(\cdot)$. Finally, we generate a well-trained encoder network $e(\cdot)$, then drop the projection head. We will use this well-trained encoder $e(\cdot)$ to generate the underlying data representations of the unlabeled dataset, then pass underlying knowledge into our UKMLP to do the proxy classification task. We will discuss the details of UKMLP in Section II-B.

Algorithm 1: **Algorithm of MedCLR.**
**INPUT:** a batch of data with size $N$, constant $\tau$, encoder $e$, projection head $p$, data augmentation module $A$
**for** sampled minibatch $\lbrace i_{k}\rbrace _{k=1}^{N}$ **do**
**for all** $k\in \lbrace 1,{\ldots },N\rbrace$ **do**
draw two augmentation functions $a\sim A$, $a^{\prime }\sim A$
# the first augmentation
$i^{\prime }_{2k-1}=a(i_{k})$ # augmentation
$r_{2k-1}=e(i^{\prime }_{2k-1})$ # representation
$z_{2k-1}=P(r_{2k-1})$ # projection
# the second augmentation
$i^{\prime \prime }_{\text{2}\;k}=a^{\prime }(i_{k})$ # augmentation
$r_{\text{2}\;k}=e(i^{\prime \prime }_{\text{2}\;k})$ # representation
$z_{\text{2}\;k}=P(r_{\text{2}\;k})$ # projection
**end for**
**for all** $n\in \lbrace 1,{\ldots },2N\rbrace$ and $m\in \lbrace 1,{\ldots },2N\rbrace$ **do**
$s_{n,m}=z_{n}^\top z_{m}/(\parallel z_{n}\parallel \parallel z_{m}\parallel)$ # pairwise similarity
**end for**
define $\ell (i^{\prime }, i^{\prime \prime })$ as $\ell (i^{\prime }, i^{\prime \prime })=-\log \frac{\exp (\operatorname{sim}(\boldsymbol{z}^{\prime }, \boldsymbol{z}^{\prime \prime }) / \tau)}{\sum _{k=1}^{\text{2}\;N} \mathbb {1}_{[k \ne i]} \exp (\operatorname{sim}(\boldsymbol{z}_{i}, \boldsymbol{z}_{k}) / \tau)}$

$\mathcal {L}=\frac{1}{\text{2}\;N} \sum _{k=1}^{N}[\ell (2 k-1,2\;k)+\ell (\text{2}\;k, 2 k-1)]$

update networks $e$ and $p$ to minimize $\mathcal {L}$
**end for**
**return** encoder network $e(\cdot)$, and throw away projection head $p(\cdot)$

### B. Underlying Knowledge Based Multi-Layer Perceptron Classifier (UKMLP)

**Figure 4.**
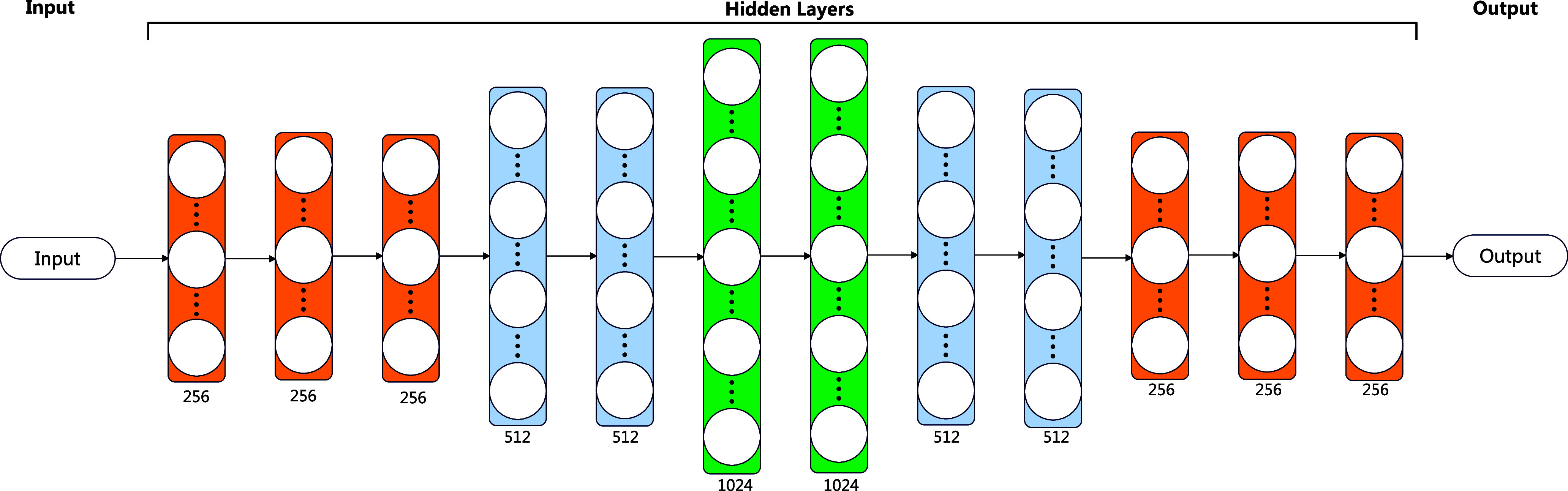
Architecture of the UKMLP. The input of UKMLP is the feature representation learned by the MedCLR, and the output is the classification result. As the figure shows, the layers with different colors represent different sizes of the layer: the orange layer contains 256 neurons, the blue layer contains 512 neurons, and the green layer contains 1024 neurons.

The UKMLP aims to fine-tune the feature representation learned by the MedCLR by the limited labeled data. This process is similar to the transfer learning to fine-tune the last layer to obtain the results, but instead, we extend the traditional Multi-layer Perceptron classifier with deeper architecture, which includes 12 hidden layers, the first three layers contain 256 neurons, then connected with two layers with 512 neurons, followed by two layers with 1024 neurons, then the size of the two layers decreases to the 512, and finally, three layers with size 256 are connected. The overall architecture is described in Fig. 4. The architecture includes three parts, the input, hidden layers, and output layer. The input comes from well-trained MedCLR, then passing the underlying knowledge into the hidden layers. The number of neurons in the output layer varies to the classes of the dataset. For each hidden layer, it follows a rectified linear activation function (ReLU), as (12) shows. The output of the ReLU function is zero if the input $x$ is lower than zero, and the value is the input value if the $x$ is larger than zero.

\begin{equation*}
 f(x) = \max(0,x) \tag{12}
\end{equation*}

The loss function of UKMLP is multi-class entropy which is shown in (13), where $\hat{y}$ is a vector of predicted class probabilities for each of the $C$ classes, $y$ is a one-hot encoded vector of the true class label, and the $\log$ is the natural logarithm.

\begin{equation*}
\mathcal {L}(\hat{y}, y) = - \sum _{i=1}^{C} y_{i} \log (\hat{y}_{i}) \tag{13}
\end{equation*}

### C. Materials and Experimental Settings

We evaluate our framework on two different medical datasets: LC25000 (https://www.kaggle.com/datasets/andrewmvd/lung-and-colon-cancer-histopathological-images), and BCCD Dataset (https://github.com/Shenggan/BCCD_Dataset). The LC25000 dataset includes five types of lung and colon cancers: Lung benign tissue, Lung adenocarcinoma, Lung squamous cell carcinoma, Colon adenocarcinoma, and Colon benign tissue, in a total of 25000 images. The BCCD dataset contains four different types of blood cell, which are Eosinophil, Lymphocyte, Monocyte and Neutrophil, in total, 12500 images. To avoid data leakage, we initially split the entire dataset with 80% of the training dataset and 20% of the test dataset. Then the training dataset splits with different labeled ratios into two datasets: unlabeled training dataset and labeled training dataset. All the images on the test dataset have ground-truth labels. So the test dataset is yet to be seen by the UKSSL before evaluating the performance of UKSSL at the last stage.

We list the hyper-parameters of our experiments in Table 1, and we run our experiments with an NVIDIA TESLA P100 16 GB RAM GPU and a Xeon CPU with 13 GB RAM. The code is implemented in Keras [36] with scikit-learn [37].

**TABLE 1:** Hyper-Parameters of Experiment

Name	Value
image size	$96 \times 96 \times 3$
epochs	200
batch size	500
temperature	0.1
patch size	6
projection dimension	64
input width of projection head	128

## III. Results and Discussion

The overall performance of our UKSSL is shown in Tables 2 and 3. In these tables, we present the performances of different datasets regarding different labeled ratios: 10%, 25%, and 50%. As Tables 2 and 3 show that our UKSSL gets a precision of 91.5%, a recall of 90.8%, an F1-score of 90.7%, and an accuracy of 90.6% if we only use 10% of the LC25000 dataset. By increasing the labeled ratio to 25%, the precision, recall, F1-score, and accuracy are 96.3%, 96.3%, 96.3%, and 96.3%, respectively. If the labeled ratio is 50%, the precision, recall, F1-score, and accuracy are 98.9%, 98.9%, 98.9%, and 98.9%, separately. We also test the performance of our method on the BCCD dataset, and it also gets good performance which shows in Table 3.

**TABLE 2:** Comparison With State-of-the-Art Methods on the LC25000 Dataset

Author	Method	labeled ratio	Precision	Recall	F1-score	Accuracy
Bukhari et al. [46]	RESNET50	100%	95.74%	81.82%	96.26%	93.91%
	RESNET18	100%	93.04%	84.21%	95.79%	93.04%
	RESNET34	100%	93.04%	80.95%	95.74%	93.04%
Phankokkruad [45]	Ensemble	100%	92%	91%	91%	91%
	ResNet50V2	100%	91%	90%	90%	90%
Hlavcheva et al. [44]	CNN-D	100%	-	-	-	94.6%
Hatuwal and Thapa [43]	CNN	100%	97.33%	97.33%	97.33%	97.2%
Mangal et al. [42]	Shallow-CNN	100%	-	-	-	97.89%
Masud et al. [47]	DL-based CNN	100%	96.39%	96.37%	96.38%	96.33%
**Ours**	**UKSSL**	10%	91.5%	90.8%	90.7%	90.6%
		25%	96.3%	96.3%	96.3%	96.3%
		**50%**	**98.9%**	**98.9%**	**98.9%**	**98.9%**

**TABLE 3:** Comparison With State-of-the-Art Methods on the BCCD Dataset

Author	Method	labeled ratio	Precision	Recall	F1-score	Accuracy
Yao et al. [41]	TWO-DCNN	100%	91.6%	91.6%	91.6%	-
Edi Jaya et al. [40]	NM-BPNN	100%	-	-	-	73.4%
Li and Chen [39]	RF	100%	-	-	78%	74.3%
Şengür et al. [38]	LSTM-based method	100%	62.36%	68.25%	64.74%	92.89%
**Ours**	**UKSSL**	10%	70.1%	67.7%	66.3%	69.1%
		25%	87.3%	86.8%	86.5%	86.2%
		**50%**	**94.3%**	**94.5%**	**94.3%**	**94.1%**

We compare the performance of our methods with other state-of-the-art methods in Tables 2 and 3. As shown in Table 2, our method improves at least 1.57% of precision, recall, F1-score, and 1.01% of accuracy when compared with other state-of-the-art methods on the LC25000 dataset, and we only use 50% labeled data to achieve the best performance. If we only use 25% labeled data, it also gets a great performance with 96.3% of precision, recall, F1-score, and accuracy, respectively. In Table 3, we show the performance of our method on the BCCD dataset, and our method improves by at least 2.7% in precision, F1-score, 2.9% in recall, 1.21% in accuracy, and we only use 50% of labeled data to get the best performance compared with other existing methods.

Table 4 shows the ablation study of our UKSSL, the ablation study uses 50% labeled datasets, and there are significant improvements in accuracy after adding the UKMLP in our UKSSL, the accuracy improves by 5.34% on the LC25000 and 30.76% on the BCCD dataset. Compared with other state-of-the-art methods, we found that UKSSL-MedCLR trained with 50% labeled LC25000 gets an accuracy of 93.56%, which means that the feature obtained by the MedCLR without fine-tuning also can achieve great performance. In this setting, the accuracy is better than the RESNET18 and RESNET34 proposed by Bukhari et al. [46], also it is better than the Ensemble and ResNet50V2 designed by the Phankokkruad [45], although all these methods use 100% labeled data. If we add UKMLP, we find that the performance is better than all the methods mentioned in Table 2. For the BCCD dataset, the UKSSL-MedCLR trained with 50% labeled data does not get a great performance, but if we add the UKMLP, the overall performance also can achieve the best accuracy compared with other methods, as Table 3 shows. The different performances trained on these two datasets are related to the complexity of the feature map in each dataset, we can easily find that the overall performance of using the LC25000 dataset is better than the BCCD dataset, as demonstrated in Tables 2 and 3, respectively. So it is acceptable that the feature map extracted by the MedCLR on the BCCD dataset is not good as LC25000 dataset. But on the other hand, we find that the UKMLP indeed helps UKSSL to get better performance.

**TABLE 4:** Ablation Study of UKSSL

Dataset (50%)	UKSSL-MedCLR	UKSSL-UKMLP	Accuracy
LC25000	✓	✗	93.56%
	✓	✓	98.9%
BCCD	✓	✗	63.34%
	✓	✓	94.1%

Although we reached outstanding performance on the different medical datasets compared with other state-of-the-art methods, there are still some challenges that need to solve. Firstly, since the data augmentation techniques are essential in both supervised and unsupervised representation learning methods [23], [24], most works consider them to define the contrastive prediction task by implementing the traditional augmented techniques. For example, our experiments use rescaling, random flipping, random translation, random zoom, and random color affine transformation. However, some traditional data augmentation techniques might not help models extract underlying knowledge from medical images. For example, some flipped medical images still look the same because the entire image with the same patterns. It is unlike the natural image, and flipping it will make huge differences compared with the original image. So it is important to use different data augmentation techniques, especially for medical images, such as contrast enhancement [22] and gamma correction [21]. Secondly, our methods highly rely on the effectiveness of data augmentation methods. Apart from designing contrastive prediction tasks by different augmented views, some works focus on modifying the model architecture, such as DIM [20] and AMDIM [19]. In the future, if the performance of our method is limited to the effectiveness of data augmentation methods, we can also try designing contrastive prediction tasks with model architectures. The last limitation of our method is computational power. Due to the limited computational power, our LTrans can only have one transformer layer, although it also can achieve good performance. At the same time, our batch size is limited to 500. We believe our framework can reach higher performance by simply expanding the size of the LTrans and other related parameters.

Our proposed UKSSL has the potential to make a significant impact in the clinical and healthcare domain. On the one hand, our method can involve different categorizations of medical images such as X-rays, CT scans, MRI scans, and histopathological slides into different classes, aiding in the diagnosis, prognosis, and treatment of different diseases. On the other hand, the proposed method can efficiently solve the annotation problems for medical images. Moreover, our method also applies to rare disease detection. Rare diseases only have limited data, making it much more difficult to train an accurate classifier. Our method applies to this situation by combining labeled data from common diseases and unlabeled data that may contain rare disease data. Then our proposed method can learn shared features across different diseases, enabling better detection of rare diseases.

## IV. Conclusion

Supervised pre-training on the large dataset and fine-tuning medical image analysis has achieved great success. Our research investigates an alternative way that uses contrastive learning to pre-train on the unlabeled medical dataset and fine-tune it with the limited labeled dataset, which constructs a semi-supervised paradigm. The proposed UKSSL includes two components: MedCLR and UKMLP. The MedCLR can extract the underlying knowledge from the unlabeled dataset, and UKMLP will fine-tune with the limited labeled dataset to achieve excellent classification performance. We get the best performance by comparing it with other state-of-the-art methods when using a limited labeled dataset, and our performance is possibly further improved by increasing the number of parameters or the size of our UKSSL. In the future, we will evaluate our method with powerful computational power and apply different contrastive learning-based methods to extract underlying knowledge from the unlabeled dataset.
